# Homelessness and Public Health: A Focus on Strategies and Solutions

**DOI:** 10.3390/ijerph182111660

**Published:** 2021-11-06

**Authors:** David A. Sleet, Louis Hugo Francescutti

**Affiliations:** 1 School of Public Health, San Diego State University, San Diego, CA 92182, USA; davidasleet@gmail.com; 2 Veritas Management, Inc., Atlanta, GA 30324, USA; 3 School of Public Health, University of Alberta, Edmonton, AB T6G 1C9, Canada; 4 Faculty of Medicine & Dentistry, University of Alberta, Edmonton, AB T6G 1C9, Canada; 5 Royal Alexandra Hospital, Edmonton, AB T5H 3V9, Canada

On any given night, hundreds of thousands of people are homeless in the United States and Canada. Globally, the problem is many times worse, making homelessness a global public health and environmental problem. The facts [1] are staggering:

- On a single night in January 2020, 580,466 people (about 18 out of every 10,000 people) experienced homelessness across the United States—a 2.2% increase from 2019.
- While 61% percent of the homeless were staying in sheltered locations, the remainder—more than 226,000 people—were in unsheltered locations on the street, in abandoned buildings, or in other places not suitable for human habitation.
- Homelessness has increased in the last four consecutive years.
- The increase in unsheltered homelessness is driven largely by increases in California.
- In 2020, 171,575 people in families with children experienced homelessness on a single night.
- A total of 3598 homeless people were children under the age of 18 without an adult present.
- Veterans comprised 8% of all homeless adults (over 46,000 veterans struggle with homelessness).
- People of color are significantly over-represented among those experiencing homelessness.

A layman’s definition of homelessness is usually “a person that has no permanent home”. However, many scholars have divided the broad group of people characterized as homeless into three (or more) categories:

- People without a place to reside;
- People in persistent poverty, forced to move constantly, and who are homeless for even brief periods of time;
- People who have lost their housing due to personal, social, or environmental circumstances.

While this definition refers specifically to homeless individuals, it is equally applicable to homeless families.

Homelessness is closely connected to declines in physical and mental health. Homeless persons experience high rates of health problems such as Human Immunodeficiency Virus/Acquired Immunodeficiency Syndrome (HIV/AIDS) and Hepatitis A infections, alcohol and drug addiction, mental illness, tuberculosis, and other serious conditions. The health problems facing homeless persons result from various factors, including a lack of housing, racism and discrimination, barriers to health care, a lack of access to adequate food and protection, limited resources for social services, and an inadequate public health infrastructure. Legal and policy interventions have often been used to attempt to address homelessness, although not always from a public health perspective. 

In health care, for example, if someone experiencing homelessness comes to an emergency department for medical aid, once treated, the only alternative is to release the patient back onto the street. This creates an endless cycle of emergency department visits, increasing costs and expending resources in the health care system.

Recent work [2] has emphasized the important role of public health, the health care system, and health care providers in homelessness prevention. In this Special Issue of the *International Journal of Environmental Research and Public Health* (IJERPH), we have brought together researchers, practitioners, and community organizers to articulate the public health problem of homelessness and identify clear strategies to reduce homelessness and provide more adequate health care and housing for this population. We also explore solutions for important subpopulations, including adults, families with children, adolescents, women, transitional aged youth, and those suffering from mental illness, PTSD, alcohol dependency, mental illness, adverse childhood experiences, and chronic homelessness.

We address many of these issues in the context of public health and explore the public health implications and potential solutions to homelessness, focusing on contemporary and emerging research and innovative strategies, and highlighting best practices to address homelessness among key populations. The papers in this Special Issue attempt to answer several questions related to homelessness and public health, such as:

- What is the extent of homelessness and why do people become homeless?
- What are the public health and health services implications of homelessness?
- What role does housing play as a precursor to and potential solution for homelessness?
- What public health and health care interventions are being employed, and what effectiveness is being achieved?
- What long-term strategies can be developed to prevent homelessness?

The 13 research papers and one commentary in this Special Issue are summarized as follows:

1. **Conceptualizing an Interdisciplinary Collective Impact Approach to Examine and Intervene in the Chronic Cycle of Homelessness.** This study by Abdel–Samad et al. [3] focuses on a novel, interdisciplinary academic–practice partnership model for addressing the problem of homelessness. Whereas singular disciplinary approaches may fall short in substantially reducing homelessness, this approach draws from a collective impact model that integrates discipline-specific approaches through mutually reinforcing activities and shared metrics. The paper describes what is necessary for capacity-building at the institution and community levels, the complementary strengths and contributions of each discipline in the model, and future implementation goals to address homelessness in the Southern California region using a cross-disciplinary approach.
2. **Mental Illness and Youth-Onset Homelessness: A Retrospective Study among Adults Experiencing Homelessness.** Iwundu et al. [4] conducted a retrospective study and evaluated the association between the timing of homelessness onset (youth versus adult) and mental illness. The results indicated that mental illness (as a reason for current homelessness) and severe mental illness comorbidities were each associated with increased odds of youth-onset homelessness, providing a basis for agencies that serve at-risk youth in order to address mental health precursors to youth homelessness.
3. **Well-Being without a Roof: Examining Well-Being among Unhoused Individuals Using Mixed Methods and Propensity Score Matching.** Ahuja et al. [5] found that the mean overall well-being score of unhoused participants was significantly lower than that of matched housed participants, with unhoused participants reporting lower mean scores for social connectedness, lifestyle and daily practices, stress and resilience, emotions, physical health, and finances. The unhoused participants had a statistically significantly higher mean score for spirituality and religiosity than their matched housed counterparts. The qualitative interviews highlighted spirituality and religion as a coping mechanism for the unhoused.
4. **Combatting Homelessness in Canada: Applying Lessons Learned from Six Tiny Villages to the Edmonton Bridge Healing Program.** Authors Wong et al. [6] discuss the Bridge Healing Program in Edmonton, Alberta, a novel approach to combatting homelessness by using hospital emergency departments (ED) as a gateway to temporary housing. The program provides residents with immediate temporary housing before transitioning them to permanent homes. The paper discusses effective strategies that underlie the Tiny Villages concept by analyzing six case studies and applying the lessons learned to improving the Bridge Healing Program and reducing repeat ED visits and ED lengths of stay among homeless individuals.
5. **Change in Housing Status among Homeless and Formerly Homeless Individuals in Quebec, Canada: A Profile Study.** Kaltsidis et al. [7] used a cluster analysis to develop a typology of the housing status change for 270 currently or formerly homeless individuals who were residing in shelters and temporary or permanent housing. The findings suggest that the maintenance or improvement in the housing status requires the availability of suitable types and frequencies of service use (enabling factors) that are well-adapted to the complexity of health problems (needs factors) among homeless individuals. Specific interventions, such as outreach programs and case management, are prioritized as necessary services, especially for individuals at a higher risk of returning to homelessness.
6. **Urban Stress Indirectly Influences Psychological Symptoms through Its Association with Distress Tolerance and Perceived Social Support among Adults Experiencing Homelessness.** To investigate the simultaneous impact of intrapersonal characteristics (distress tolerance) and interpersonal characteristics (social support) and their association with homelessness, Hernandez et al. [8] recruited homeless adults from six homeless shelters in Oklahoma City who self-reported urban life stress, distress tolerance, social support, major depressive disorder, and PTSD symptoms. Based on the resulting associations, their findings stress the importance of implementing interventions aimed at increasing social support for homeless persons, something that may also increase skill development for distress tolerance and indirectly lead to a reduction in depression and PTSD.
7. **“I Felt Safe”: The Role of the Rapid Rehousing Program in Supporting the Security of Families Experiencing Homelessness in Salt Lake County, Utah.** Garcia and Kim [9] describe their research into The Road Home (TRH) program, which provides services to homeless individuals and families. TRH is known for their emergency shelters and also administers the Rapid Rehousing Program (RRHP), designed to help homeless families transition back into stable housing. After collecting qualitative data from focus groups with participants and families, landlords, case managers, and service providers, they make recommendations for program improvements that can increase the residential security of families experiencing homelessness.
8. **“It’s Just a Band-Aid on Something No One Really Wants to See or Acknowledge”: A Photovoice Study with Transitional Aged Youth Experiencing Homelessness to Examine the Roots of San Diego’s 2016–2018 Hepatitis A Outbreak.** In this study, Felner et al. [10] examined the experiences and needs of transitional aged youth (TAY) aged 18–24 experiencing homelessness who may have been uniquely affected by an unprecedented outbreak of hepatitis A virus (HAV). The findings documented a stigmatization of TAY, interventions that failed to address root causes of the outbreak, and interactions with housing- and social support-related resources that limited rather than supported economic and social mobility. The findings have implications for understanding how media and public discourse, public health interventions, and the availability and delivery of resources can contribute to and perpetuate stigma and health inequities faced by TAY experiencing homelessness.
9. **Predictors of Overnight and Emergency Treatment among Homeless Adults.** Iwundu et al. [11] aimed to identify the sociodemographic predictors associated with overnight and emergency hospital treatment among a sample of homeless adults. Participants were recruited from a shelter in Dallas, Texas and were predominantly uninsured, low-income men and women from various social and ethnic groups. In logistic regression models, gender emerged as the only predictor of overnight treatment in a hospital and treatment in an emergency department. Women were more likely than men to be treated overnight and use emergency care. The authors concluded that interventions and policies targeted toward homeless women’s primary health care needs would reduce health care costs.
10. **Association of Problematic Alcohol Use and Food Insecurity among Homeless Men and Women.** In a study on alcohol use and food insecurity among homeless men and women, Reitzel et al. [12] investigated the link between problematic alcohol use and food insecurity among homeless adults in Oklahoma. Problematic alcohol use was measured using the Alcohol Quantity and Frequency Questionnaire and the Patient Health Questionnaire. Food insecurity was measured with the USDA Food Security Scale-Short Form. The results indicated that heavy drinking and probable alcohol dependence/abuse were each associated with increased odds of food insecurity. The results question whether alcohol may take precedence over eating or food purchases among this population of homeless individuals.
11. **Exploring Tiny Homes as an Affordable Housing Strategy to Ameliorate Homelessness: A Case Study of the Dwellings in Tallahassee, FL.** “Tiny Homes” is an emerging strategy to combat homelessness, and Jackson et al. [13] raise a number of questions about the intentions, efficacy, and policy feasibility of this strategy. The paper seeks to understand the strategies used by stakeholders to plan, design, and implement a “Tiny Homes” strategy, and to assess their effectiveness. Using a case study, they examined how the community was planned, the experiences of residents, and the constraints to success. Their findings highlighted how funding constraints and NIMBYism (Not in My Backyard-ism) stymied stakeholder efforts to achieve equity and affordability, resulting in the inability to achieve project aims to develop affordable housing that served homeless populations.
12. **Predictors of Emergency Department Use among Individuals with Current or Previous Experience of Homelessness.** The study by Gabet et al. [14] assessed the contributions of predisposing, enabling, and needs factors in predicting emergency department (ED) use among 270 individuals with a current or previous experience of homelessness. Participants were recruited from types of housing in Montreal, Quebec (Canada) and were interviewed about their ED use at baseline and again 12 months later. The findings revealed two needs factors associated with ED use: having a substance use disorder and low perceived physical health. Two enabling factors—the use of ambulatory specialized services and stigma—were also related to ED use. ED use was not associated with the type of housing. The authors suggest that improvements are needed to manage substance use disorders and the physical health of homeless individuals in order to reduce ED use.
13. **Being at the Bottom Rung of the Ladder in an Unequal Society: A Qualitative Analysis of Stories of People without a Home.** The Mabhala and Yohannes article [15] examines the stories of homeless people and their perceptions of their social status using interviews in three centers for homeless people in Cheshire, in the English Northwest. Education, employment, and health were three domains that provided a theoretical explanation for the reasons that led to their homelessness. Participants catalogued their adverse childhood experiences, which they believe limited their capacity to meaningfully engage with social institutions for social goods, such as education, social services, and institutions of employment. They conclude that, although not all people who are poorly educated, in poor health, and unemployed end up being homeless, a combination of these together with multiple adverse childhood experiences may weaken resilience and contribute to homelessness.
14. **Commentary: Investing in Public Health Infrastructure to Address the Complexities of Homelessness.** In a final commentary, Allegrante and Sleet [16] introduce the notion that investments in public health infrastructure are needed to address the complexities of homelessness, including the continued threats posed by SARS-CoV-2 (COVID-19) and its variants. The lack of affordable housing, widespread unemployment, poverty, addiction and mental illness, which all contribute to the risk of homelessness, would be well-served by improving the fundamental public health infrastructure. They argue that homelessness is exacerbated by system-wide infrastructure failures at the municipal, state and federal governments and from the neglect to invest in public infrastructure, including a modern public health system.

In conclusion, shelter is a basic human need. Thus far, we have an inadequate understanding of all the medical and nonmedical, public health, and infrastructural influences that drive homelessness and why so many people are living without adequate shelter. Housing is one of the most critical factors in addressing homelessness and one of the best-researched social determinants of health. Several articles here focus on innovative approaches to providing temporary or permanent housing for those who need it, and it is well known that selected housing interventions can improve health and decrease health care costs. From that perspective, some professionals in the field contend that housing equates to health [17] and that improved housing options for homeless individuals and families would advance population-level health.

Many of the articles in this Special Issue [18] focus on specific aspects of life, quality of life, and co-morbidities related to behavioral and social variables influencing homelessness. Explored in detail are factors such as lack of housing, distress, wellness, emergency department use, mental health, drug and alcohol addiction, poverty, low educational attainment, inadequate health care and social services, adverse childhood experiences, ongoing infections, unemployment, and public health infrastructure. In addition to highlighting the impact these factors can have on the likelihood that someone would become homeless, many of the articles also provide recommendations for relevant policies, practices, and interventions that could help reduce homelessness and improve overall well-being.

The intersection of environmental, behavioral, and social factors, in addition to the lack of an adequate infrastructure, must also be considered when studying the determinants of homelessness and designing appropriate interventions. Our ultimate goal in producing this Special Issue of *IJERPH* is to encourage the development of better evidence to inform public health, social services, and medical care policies and practices that will result in better health for homeless populations.
